# Increased interleukin-6 and macrophage chemoattractant protein-1 are associated with respiratory failure in COVID-19

**DOI:** 10.1038/s41598-020-78710-7

**Published:** 2020-12-10

**Authors:** Marthe Jøntvedt Jørgensen, Jan Cato Holter, Erik Egeland Christensen, Camilla Schjalm, Kristian Tonby, Søren Erik Pischke, Synne Jenum, Linda G. Skeie, Sarah Nur, Andreas Lind, Hanne Opsand, Tone Burvald Enersen, Ragnhild Grøndahl, Anne Hermann, Susanne Dudman, Fredrik Muller, Thor Ueland, Tom Eirik Mollnes, Pål Aukrust, Lars Heggelund, Aleksander Rygh Holten, Anne Ma Dyrhol-Riise

**Affiliations:** 1 Department of Infectious Diseases, Oslo University Hospital, Oslo, Norway; 2 Institute of Clinical Medicine, University of Oslo, Oslo, Norway; 3 Department of Microbiology, Oslo University Hospital, Oslo, Norway; 4 Department of Immunology, Oslo University Hospital, Oslo, Norway; 5 Division of Emergencies and Critical Care, Oslo University Hospital, Oslo, Norway; 6 Department of Internal Medicine, Drammen Hospital, Vestre Viken Hospital Trust, Drammen, Norway; 7 Department of Rheumatology, Drammen Hospital, Vestre Viken Hospital Trust, Drammen, Norway; 8 Department of Laboratory Medicine, Drammen Hospital, Vestre Viken Hospital Trust, Drammen, Norway; 9 Research Institute of Internal Medicine, Oslo University Hospital, Rikshospitalet, Oslo, Norway; 10 Research Laboratory, Nordland Hospital, Bodø, Norway; 11 Faculty of Health Sciences, K.G. Jebsen TREC, University of Tromsø, Tromsø, Norway; 12 Centre of Molecular Inflammation Research, Norwegian University of Science and Technology, Trondheim, Norway; 13 Department of Clinical Science, University of Bergen, Bergen, Norway; 14 Department of Acute Medicine, Oslo University Hospital, Oslo, Norway; 15 Section of Clinical Immunology and Infectious Diseases, Oslo University Hospital, Oslo, Norway

**Keywords:** Cytokines, Infectious diseases

## Abstract

In SARS-CoV-2 infection there is an urgent need to identify patients that will progress to severe COVID-19 and may benefit from targeted treatment. In this study we analyzed plasma cytokines in COVID-19 patients and investigated their association with respiratory failure (RF) and treatment in Intensive Care Unit (ICU). Hospitalized patients (n = 34) with confirmed COVID-19 were recruited into a prospective cohort study. Clinical data and blood samples were collected at inclusion and after 2–5 and 7–10 days. RF was defined as PaO2/FiO2 ratio (P/F) < 40 kPa. Plasma cytokines were analyzed by a Human Cytokine 27-plex assay. COVID-19 patients with RF and/or treated in ICU showed overall increased systemic cytokine levels. Plasma IL-6, IL-8, G-CSF, MCP-1, MIP-1α levels were negatively correlated with P/F, whereas combinations of IL-6, IP-10, IL-1ra and MCP-1 showed the best association with RF in ROC analysis (AUC 0.79–0.80, *p* < 0.05). During hospitalization the decline was most significant for IP-10 (*p* < 0.001). Elevated levels of pro-inflammatory cytokines were present in patients with severe COVID-19. IL-6 and MCP-1 were inversely correlated with P/F with the largest AUC in ROC analyses and should be further explored as biomarkers to identify patients at risk for severe RF and as targets for improved treatment strategies.

## Introduction

Severe Acute Respiratory Syndrome coronavirus 2 (SARS-CoV-2), identified as the causative agent of coronavirus disease (COVID-19) has quickly spread globally since first reported in December 2019^1–4^. The disease usually presents with pneumonia-like signs and symptoms^5^. Most people infected with SARS-CoV-2 experience mild to moderate disease, but some may suffer severe respiratory failure (RF) and acute respiratory distress syndrome (ARDS) in need of mechanical ventilation^5^. Clinical management of the disease is mainly supportive care, although despite limited evidence, a variety of anti-viral and immunomodulatory therapies have been suggested^6–8^.

Severe SARS-CoV-2 infection cause a dysregulated immune response resulting in excessive production of inflammatory cytokines and chemokines that contributes to the pathogenesis^9–11^. Indeed, viral evasion of initial immune responses and a subsequent immunological misfiring causing collateral tissue injury in infected organs seem to play major roles in severe COVID-19^12–15^. Further, evidence suggests that a suboptimal or inappropriate T cell response producing pro-inflammatory cytokines may contribute to tissue damage in critically ill COVID-19 patients^16,17^.

Lymphopenia, hyperferritinemia and increased D-dimer are associated with severe COVID-19^12,18^. Reports suggests that the hyperinflammation seen in severe COVID-19 may be driven by considerable levels of C-reactive protein (CRP) and Interleukin (IL)-6, resembling “cytokine storms” seen in other comparable conditions such as during CAR T cell therapy^19–21^. Thus, treatment with immunomodulatory therapies targeting IL-6 or IL-1 receptors could possibly also be of value in COVID-19^22–24^.

Deeper understanding of the pathogenesis of severe COVID-19 is of major importance for the development of targeted immune therapy. There is also an urgent need for prognostic biomarkers in order to early identify patients that will progress into critical COVID-19. Inflammatory mediators are operating in a complex network and the aim of the present study was to map and characterize this network, including interleukins, interferons, chemokines and growth factors, in plasma collected from patients with various severity of confirmed COVID-19. We explored their potential as prognostic biomarkers for RF, need of treatment in an intensive care unit (ICU) and their relation to routine biochemical and hematological markers of hyperinflammation.

## Methods

### Study design and population

Adult patients (≥ 18 years old) hospitalized between March 6th and April 14th with confirmed SARS-CoV-2 infection by positive real-time polymerase chain reaction test targeting the E-gene of SARS-CoV-2 were consecutively recruited from Oslo University Hospital, Ullevål and Drammen Hospital, Vestre Viken Hospital Trust, Norway to a multi-center prospective cohort study (Norwegian SARS-CoV-2 study; NCT04381819). Recent publications report on patients included in the same cohort study^25–28^. Clinical information and laboratory samples were for most cases collected within 48 h after hospitalization. Peripheral blood was drawn at inclusion, day 2–5 and day 7–10 during hospitalization.

### Data collection

Clinical and routine diagnostics were abstracted from electronic medical records using a modified version of the International Severe Acute Respiratory and emerging Infection Consortium (ISARIC, isaric.tghn.org) /World Health Organization (WHO) Clinical Characterization Protocol (CCP) and entered into a secure web-based REDCap database (Research Electronic Data Capture, Vanderbilt University, TN, hosted by University of Oxford, UK).

### Clinical outcomes

Clinical outcomes were: (1) development of respiratory failure (RF) during hospitalization defined as an arterial partial pressure of oxygen (PaO_2_) to fraction of inspired oxygen (FiO_2_) ratio (P/F) of less than 40 kPa (< 300 mm Hg), corresponding to the threshold-value for ARDS by the Berlin definition^29^, and (2) need of treatment in ICU. Where PaO_2_ was not available from the records, PaO2 values were calculated from SaO_2_^30^.

### Ethical considerations

Informed consents were obtained from all patients or patients' family members. The study was approved by the Regional Committee for Medical and Health Research Ethics in South-Eastern Norway (reference number 106624, clinicaltrial.gov NCT04381819). All methods were performed in accordance with the relevant guidelines and regulations.

### Sample processing

Peripheral blood was collected into 4 mL Vacuette^®^ (Greiner bio-one International) containing EDTA to avoid coagulation. Samples were immediately stored on ice, processed within 30 min and plasma were isolated by centrifugation at 2000*g* for 20 min at 4 °C. Plasma were immediately frozen at − 80 °C in several aliquots and thawed only once prior to multiplex analysis.

### Multiplex analyses

**Figure 1:**
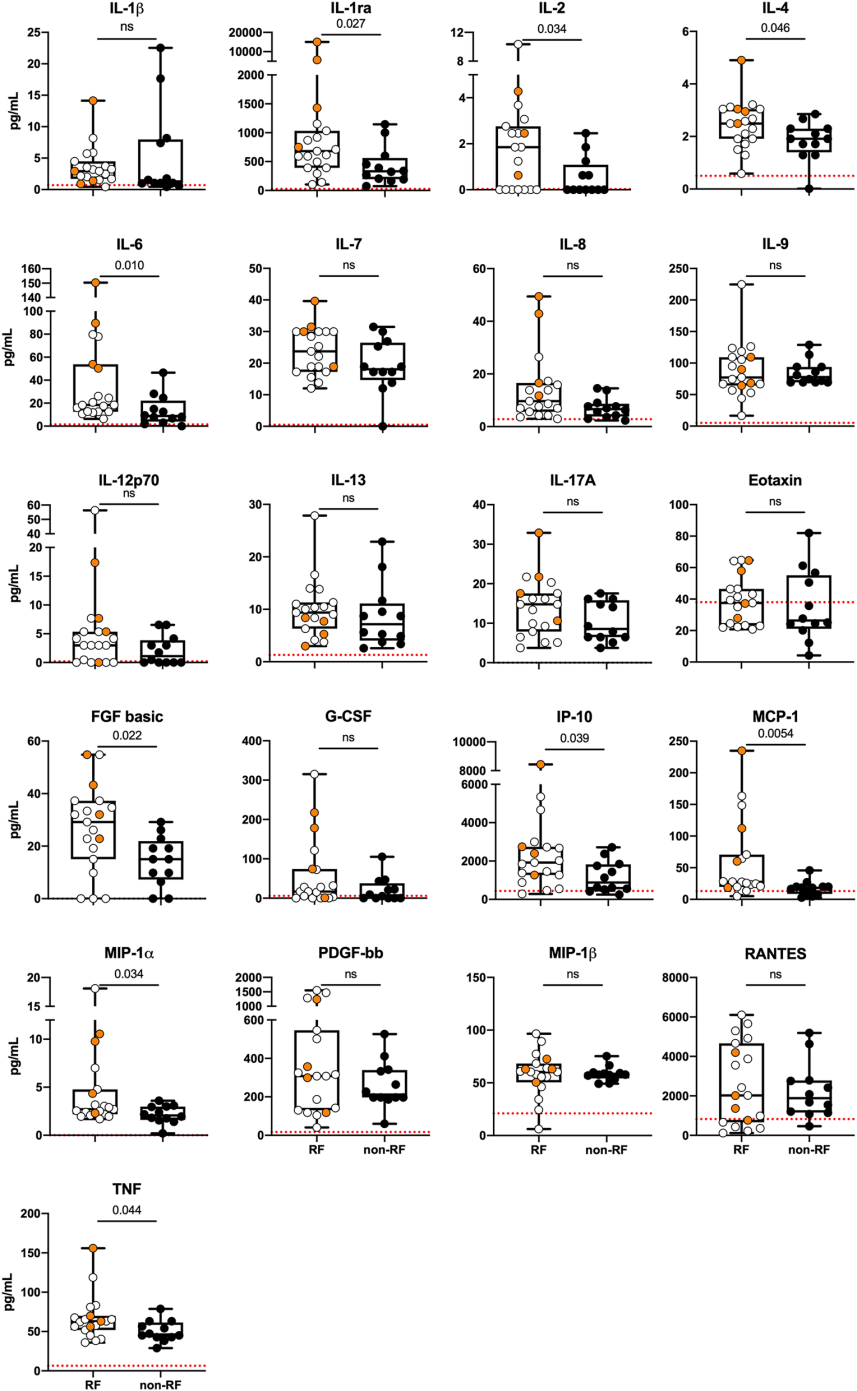
Cytokines at admission in COVID-19 patients with respiratory failure. Twenty-one cytokines had detectable levels in plasma from COVID-19 patients at admission to hospital. The levels of IL-1ra, IL-2, IL-4, IL-6, FGF basic, MCP-1, MIP-1α and TNF were significantly higher in patients with RF (n = 19) compared to non-RF patients (n = 12). For three patients baseline samples were not available and these were excluded from the analysis set. Patients that died during hospitalization (n = 4) are shown in orange circles. Red lines indicate reference values by Hennø et al.^31^. Groups were compared using Mann U Whitney test, significance * < 0.05.

EDTA plasma samples from 34 hospitalized patients (Drammen hospital [n = 16] and Oslo University Hospital Ullevål [n = 18] were analyzed using a multiplex cytokine assay (Bio-Plex Human Cytokine 27-Plex Panel; Bio-Rad Laboratories Inc., Hercules, CA) containing the following interleukins, chemokines and growth factors: Interleukin (IL)-1β, IL-1 receptor antagonist (IL1-ra), IL-2, IL-4, IL-5, IL-6, IL-7, IL-8/CXCL8, IL-9, IL-10, IL-12 (p70), IL-13, IL-15, IL-17, eotaxin/CXCL11, basic fibroblast growth factor (bFGF), granulocyte-colony stimulating factor (G-CSF), granulocyte macrophage colony stimulating factor (GM-CSF), interferon (IFN)-γ, interferon-inducible protein (IP-10)/CXCL10, monocyte chemotactic protein (MCP-1)/CCL2, macrophage inflammatory protein (MIP)-1α/CCL3, MIP-1β/CCL4, platelet derived growth factor-BB (PDGF-BB), regulated upon activation T cell expressed and secreted (RANTES)/CCL5, tumor necrosis factor (TNF), and vascular endothelial growth factor (VEGF). The samples were analyzed on a Multiplex Analyzer (Bio-Rad Laboratories) according to instructions from the manufacturer. Six of the 27 analytes were not detectable in more than 30% of the samples and were therefore excluded for further analysis (IL-5, IL-10, IL-15, GM-CSF, VEGF and INF-γ). A limited number of samples were below the lower detection limit and in the statistical analyses these were given a random number below the detection limit. The normal reference values for this 27-plax assay are indicated with red lines in Fig. 1^31^.

### Statistical analysis

Non-parametric testing was used to investigate differences between two or more groups. Mann–Whitney U test was used to compare two independent groups while Kruskal–Wallis was used comparing three groups. Friedman’s test was applied on longitudinal samples, investigating changes from baseline, day 2–5 and day 7–10. *p* values were considered significant when < 0.05. Due to the explorative nature of the study the statistical analyses were not corrected for multiple testing. The area under the receiver operating characteristics (ROC) curve (AUC) was estimated for patients with or without RF. The optimal cut-off value was defined as the value with the highest Youden’s Index. Correlations were calculated using Spearman rank correlation coefficient. Statistical analysis was performed by SPSS statistical software (Macintosh version 26.0, IBM, Armonk, NY, USA).

## Results

### Patient characteristics

Table 1 displays demographic and clinical characteristics of the COVID-19 patients included in this study (n = 34). Median age was 58 years and 74% were men. Twenty-one of the 34 patients developed RF reflected in a P/F ratio < 40 kPa. Ten patients, all with RF, were treated in the ICU including four patients that died during hospitalization. RF patients were mostly men (86%), had higher leucocyte count and higher D-dimer, ferritin, C-reactive protein (CRP) and bilirubin levels compared to the non-RF group. In contrast, the median number of days between the first onset of symptoms and admission to hospital were comparable and there were no differences in age, previous co-morbidities or kidney function (eGFR) between the two groups.

**Table 1:** Patient characteristics.

	Total (n = 34)	RF (n = 21)	Non-RF (n = 13)	ICU (n = 10)	Non-ICU (n = 24)
Age	58 (27–91)	54 (45–91)	63 (27–91)	58 (46–91)	58 (27–91)
Sex, male	25 (74)	18 (86)*	7 (54)	9 (90)	16 (68)
RF	21 (62)	21 (100)	–	10 (100)	11 (46)
ICU	11 (32)	10 (48)	0	10 (100)^**a**^	–
Days (symptom to hospital admission)	10 (2–38)	10 (2–37)	10 (3–38)	9 (2–37)	10 (3–38)
**Co-morbidities**					
Hypertension	6 (15)	2 (10)	4 (30)	0 (0)	6 (26)
Diabetes	4 (12)	2 (10)	2 (15)	1 (10)	3 (13)
Chronic heart disease	8 (24)	4 (19)	4 (31)	4 (40)	4 (17)
Chronic lung disease	1 (3)	1 (5)	0	1 (10)	0
Chronic kidney disease	4 (12)	2 (10)	2 (15)	2 (20)	2 (8)
**Symptoms and O**_**2**_** therapy**					
Fever	30 (88)	18 (86)	12 (92)	8 (80)	22 (92)
Cough	28 (82)	17 (81)	11 (85)	8 (80)	20 (83)
Sputum	11 (32)	8 (38)	3 (23)	2 (20)	9 (38)
Sore throat	10 (29)	5 (24)	5 (39)	1 (10)*	9 (38)
Dyspnoae	22 (65)	16 (76)	6 (46)	8 (80)	14 (58)
Respiratory rate > 22 (breaths per min)	22 (65)	15 (71)	7 (54)	9 (82)	12 (52)
${\text{Pa}}_{{\text{O}}_{2}}\text{/}{\text{Fi}}_{{\text{O}}_{2}}$ ratio	39 (8–66)	32 (8–45)^§^	46 (32–66)	28 (8–40)^#^	43 (23–66)
Oxygen therapy	28 (82)	20 (95)*	8 (62)	10 (100)	18 (75)
**Laboratory**					
Leucocytes (× 10^9^/L)	5.8 (2.6–19.0)	7.2 (3.8–19.0)^#^	4.9 (2.6–8.1)	9.3 (3.8–19.0)*	5.4 (2.6–12.0)
Lymphocytes (× 10^9^/L)	1.1 (0.3–2.1)	1.0 (0.5–1.7)	1.2 (0.3–2.1)	1.1 (0.5–1.5)	1.1 (0.3–2.1)
Platelets (× 10^9^/L)	192 (110–611)	201 (136–611)	186 (110–302)	192 (161–611)	193 (110–350)
Haemoglobin (g/dL)	13.4 (8.5–17.9)	13.6 (8.5–17.9)	13.2 (9.8–15.2)	12.8 (8.5–17.9)	13.4 (9.8–15.6)
D-dimer (mg/L)	0.80 (0.30–4.10)	1.2 (0.48–4.10)*	0.55 (0.30–4.0)	1.60 (0.71–4.10)*	0.76 (0.30–4.0)
Ferritin (μg/L)	814 (105–2893)	1114 (178–2893)^§^	455 (105–1609)	1226 (178–2893)	747 (105–2777)
C-reactive protein, (mg/L)	67 (3–448)	117 (23–448)*	46 (3–191)	151 (44–448)^#^	47 (3–196)
LDH (U/L)	322 (118–561)	350 (146–561)	274 (118–354)	441 (146–561)*	283 (118–479)
eGFR (mL/min/1.73 m^3^)	86 (13–164)	86 (23–164)	79 (13–113)	81 (23–164)	91 (13–120)
Bilirubin total, (μmol/L)	10 (3–17)	11 (8–17)*	8 (3–17)	12 (8–17)	9 (3–17)

Data are given as median (range), n (%).

Significance indicated with *p < 0.05, ^#^*p* < 0.01, ^§^*p* < 0.001.

^a^One patient who died was in need of ICU but was not transferred due to high age.

### Elevated cytokine levels in COVID-19 patients with respiratory failure and in ICU

**Figure 2:**
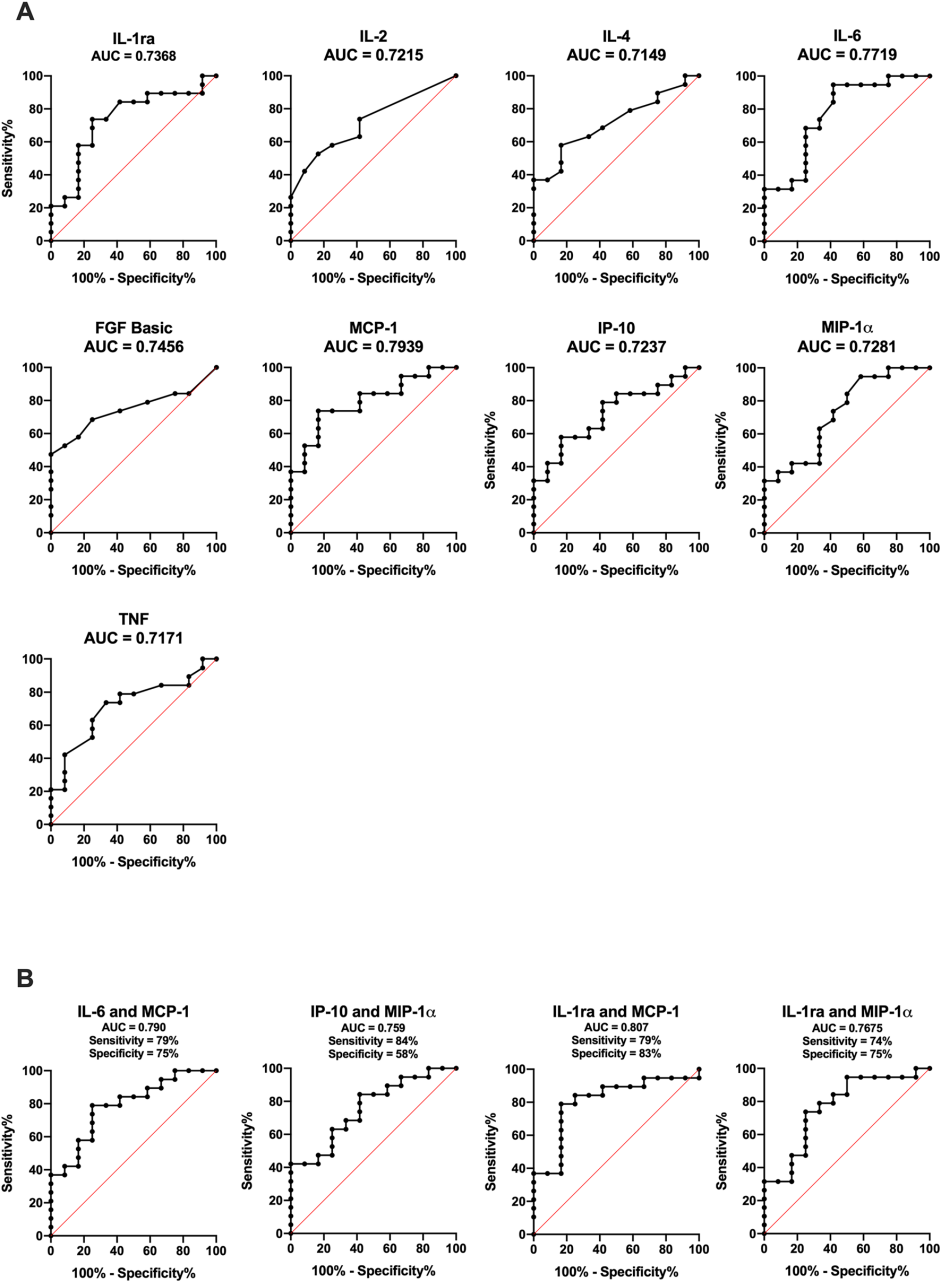
ROC analyses of cytokines in patients with respiratory failure. The receiver operating characteristic (ROC) curve was calculated comparing baseline plasma (**A**) cytokine levels and (**B**) combination of cytokine levels in RF vs non-RF patients with calculated sensitivity and specificity. Area under the ROC curve (AUROC) *p* values were < 0.05. Cytokines for ROC analyzes were selected on whether they showed significant difference between the two groups (Fig. 1).

To investigate a potential dysregulated immune response in critically ill COVID-19 patients we analyzed plasma levels of 27 cytokines. All of the 21 cytokines with reliably detectable plasma levels (see “Methods” section), showed higher levels in COVID-19 patients than in healthy controls (red line Fig. 1) obtained from Hennø et al.^31^. Nine mediators (IL-1ra, IL-2, IL-4, IL-6, FGF basic, IP-10, MCP-1, MIP-1α and TNF) were significantly higher in the RF group compared to non-RF patients at admission (Fig. 1) and IL-6 (0.50, *p* < 0.01), G-CSF (0.55, *p* < 0.01), MCP-1 (0.45, *p* < 0.05) and MIP-1α (0.49, *p* < 0.05) levels showed a negative correlation with P/F ratio. We also investigated the relationship between RF and cytokine levels by ROC analysis (Fig. 2). MCP-1 (0.79) and IL-6 (0.77) and the combinations of MCP-1/IL-6 (0.79) and MCP-1/IL-1ra 1 (0.81) demonstrated the largest, although modest, areas under the curve (AUC) (Fig. 2B). The optimal cut-off values (the highest Youden’s Index) for the cytokines are displayed in Table 2. The calculated values for IL-6 and MCP-1 in predicting RF were 10 pg/mL and 20 pg/mL respectively.

**Table 2:** Prediction of respiratory failure by plasma cytokine analyses.

	Sensitivity %	95% CI	Specificity %	95%CI	Optimal cut-off (pg/mL)
IL-1ra	74	51–88	75	47–91	474
IL-6	95	75–100	58	32–81	10
MCP-1	74	51–88	83	55–97	20
IP-10	58	36–77	83	55–97	1849
FGF basic	47	27–68	100	75–100	31

**Figure 3:**
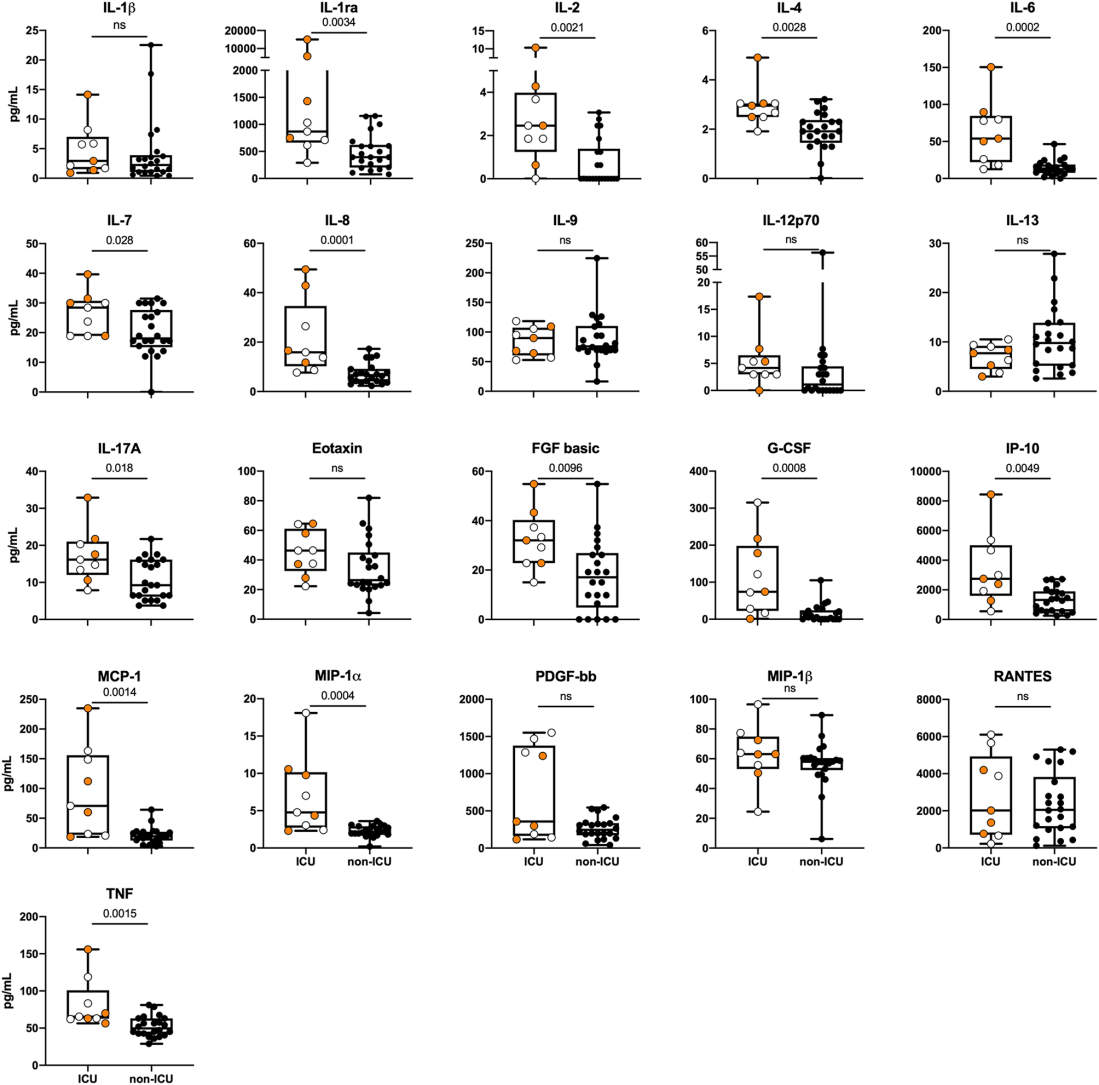
Cytokine levels in COVID-19 patients in ICU compared to non-ICU. Cytokine levels in patients with (n = 9) or without (n = 22) treatment in ICU. For three patients baseline samples were not available and these were excluded from the analysis set. Groups were compared using Mann U Whitney test, significance < 0.05.

Further, when stratifying the patients dependent on the need of treatment in an ICU compared to regular ward we found significant higher levels of altogether 13 cytokines (IL-1ra, IL-2, IL-4, IL-6, IL-7, IL-8, IL-17A, FGF basic, G-CSF, IP-10, MCP-1, MIP-1α and TNF) in ICU compared to non-ICU patients (Fig. 3).

### Relationship between biochemical and hematological markers of inflammation and cytokine levels in patients with COVID-19

**Figure 4:**
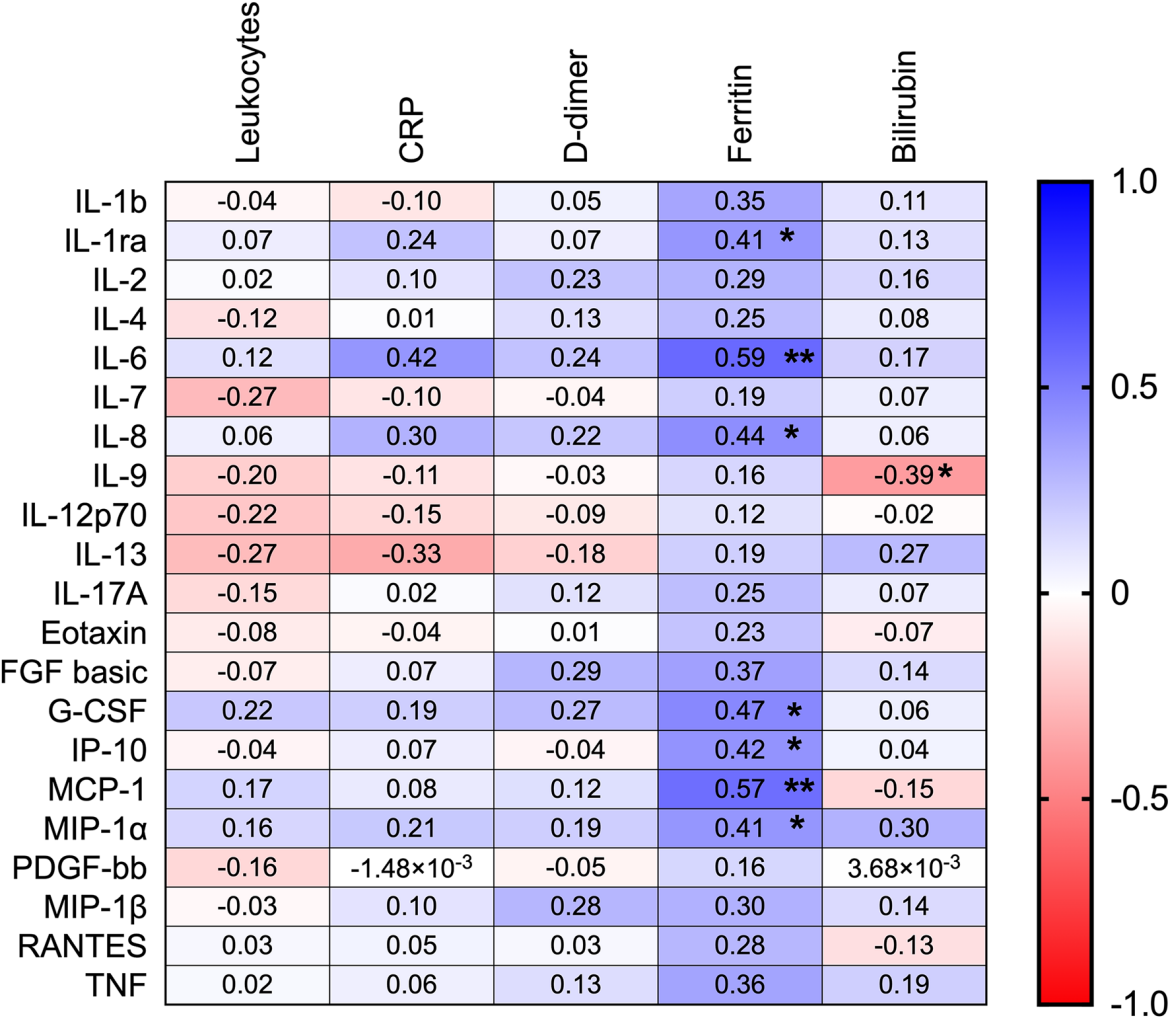
Correlation between biochemistry/hematology parameters and Cytokines. Table displaying Rho values from correlation analysis. Plasma cytokine levels were correlated to leukocyte count (10^9^/L, n = 29), CRP (mg/L, n = 29), D-dimer (n = 23), ferritin (μg/L, n = 29) and Bilirubin (μmol/L, n = 25). Correlations were calculated with Spearman correlation coefficient. Blue color indicates a positive correlation while red represents a negative correlation. Significant correlations are displayed with *. Significance * < 0.05, ** < 0.01.

Since several cytokines were significantly elevated in COVID-19 patients with RF, we further investigated if any of these cytokines were associated with other biochemical and hematological markers of inflammation (Fig. 4). IL-1ra, IL-6, IL-8, G-CSF, MCP-1, IP-10, MIP-1α showed a positive correlation with ferritin, whereas there were no correlations found between cytokines and leukocyte count, CRP or D-dimer. Only IL-9 showed a positive correlation with bilirubin.

### Dynamic changes in cytokine levels in COVID-19 patients during hospitalization

**Figure 5:**
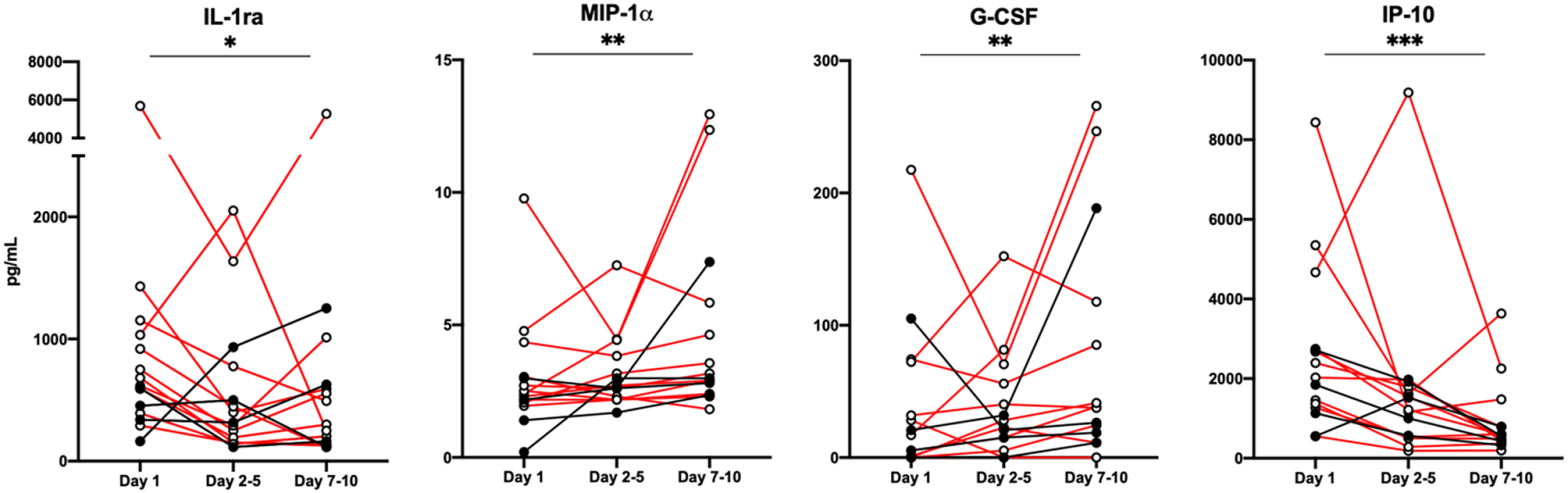
Longitudinal cytokine levels in COVID-19 patients during hospitalization. Measurement of plasma cytokine levels at day 1 (n = 15), day 2–5 (n = 15) and day 7–10 (n = 15) after hospitalization. Scatter plot displaying individual values of longitudinal data showing a significant change in the levels of four cytokines. Significance calculated with Friedmans test, * < 0.05, ** < 0.01, *** < 0.001. Patients with RF (n = 11) are shown in red.

Cytokine plasma levels were analyzed at several time points during hospitalization to explore their dynamics in COVID-19 (Supplementary Figure S1). Of the 15 patients with available longitudinal samples three were treated in ICU for the whole period, one was transferred to ICU at day 2–5, whereas another two patients were transferred to ICU after 7–10 days. Of the ten cytokines that were elevated in RF patients at baseline, only IL-1ra, MIP-1α, G-CSF and IP-10 displayed significant changes during follow-up (Fig. 5). Overall, especially IP-10 levels were reduced for the majority of patients irrespective of P/F values at the last time point (*p* < 0.001). Of note, for two of the three patients that were referred to ICU during hospitalization, there was a corresponding increase in plasma IL-1ra, MIP-1α, G-CSF and IP-10 levels.

## Discussion

We present data from a Norwegian COVID-19 patient cohort where plasma cytokine levels at admission and follow up during the first ten days of hospitalization were related to disease severity expressed as RF and/or need of ICU treatment or signs of hyperinflammation in peripheral blood. We show that a broad network of pro-inflammatory cytokines is elevated in plasma from patients with COVID-19, especially pronounced in patients with severe RF and even more obvious in patients with need of ICU care. IL-6 and MCP-1 seemed to be of particular interest since they were markedly elevated in patients with RF, significantly inversely correlated with P/F ratio and showed modest, still the largest AUC in ROC analyses. Thus, our data demonstrates a marked dysregulation of the cytokine network in COVID-19 patients and for MCP-1 and IL-6, the levels were strongly correlated with the degree of RF.

Although inflammation is a protective process to clear any foreign pathogen, excessive inflammation can cause severe collateral tissue damage. Our findings of elevated cytokine levels in patients with RF, ICU and hyperinflammation are consistent with earlier reports of SARS-CoV^10^, SARS-CoV-2^13,23,32^ and for ARDS alone^33^. The cytokine release syndrome has been suggested as an important cause of ARDS and RF in COVID-19 patients^13,22,23,34^. We demonstrated increased levels of a wide range of inflammatory cytokines in these patients such as IL-1ra, IL-6, IP-10, G-CSF, MCP-1, MIP-1α and TNF, all rapidly released upon innate immune activation and important for shaping the adaptive immune response and induce T cell activation^35^. This is supported by data from the same cohort showing that COVID-19 patients with poor outcome and cardiac involvement are characterized by activated and exhausted T cells^26^. Together this may reflect systemic cytokine activation in the RF patients, contributing to immunopathology and pulmonary dysfunction in COVID-19^14,15,23^. Of these cytokines, IL-6 and MCP-1 were consistently associated with RF. It has recently been suggested that IL-6, IL-1ra, IP-10 and MCP-3 could serve as predictive markers for disease progression in COVID-19^32,36–38^*.* Several studies have suggested a link between high IL-6 levels and disease severity in COVID-19, indicating that IL- 6 is involved in down-regulation of HLA-DR and lymphopenia, contributing to the sustained cytokine levels seen in severe COVID-19^11,15^. Data on MCP-1 levels in severe SARS-CoV suggest that secretion of MCP-1 is associated with lung injury^10,12,39^, and our finding indicates that this macrophage activating chemokine could play a pathogenic role in COVID-19 associated RF. The calculated cut-off value of IL-6 for predicting RF (10 pg/mL) in our study was lower than the predictive value of 65 pg/mL in a study of a combined evaluation and validation cohort^37^ and in another study predicting mortality (86 pg/mL)^40^. The different values could be due to different definitions and measurements of RF. Herold et al*.,* used the need for mechanical ventilation as a measure of RF^37^ whereas P/F ratio less than 40 kPa was defined as RF in our study. These studies, including ours have a relatively low number of patients that progress to severe disease, and larger cohorts are needed to validate the predictive cut-off values for IL-6 for various clinical outcomes.

In addition to IL-6 and MCP-1, we show that the combinations of these cytokines with IL-1ra and IP-10 were equally good as predictors of COVID-19 severity. IL-1ra circulates in much higher levels than IL-1 itself, is involved in NLRP3 inflammasome activation and has been suggested as a reliable marker of IL-1 activity in COVID-19 disease^41^. IP-10 is an important mediator of monocyte/macrophage-induced T cell activation also proposed to play a role in COVID-19 pathogenesis^36,42,43^. IP-10 is markedly elevated both in blood and lung tissue from SARS-patients^44^. We found that the marked elevation of IP-10 levels correlating with COVID-19 severity declined throughout hospitalization, in line with a recently published study and in previous studies in SARS^45^. Interestingly, there was a corresponding increase in not only IP-10, but also IL-1ra, MIP-1a and G-CSF in patients transferred to ICU during hospitalization indicating a worsening of clinical condition.

Despite that plasma inflammatory cytokines are highly heterogeneous with a wide range of biological functions they serve as valuable biomarkers in the diagnosis, management and prognosis of several inflammatory diseases^46^. We suggest that IL-6 and MCP-1 should be added to the list of candidate markers for disease prediction in COVID-19 patients with RF, as well as be further explored as targets for immune therapy with cytokine antagonists. Still, although the levels of pro-inflammatory cytokines seem to be increased in patients with severe COVID-19, the correlation between cytokines and clinical stage are far from consistent. Early reports argue that lower levels of IL-6 were observed in severe COVID-19, inconsistent with a true cytokine storm^47–49^. However, emerging evidence shows that IL-6 seems to play a central role in cytokine storm reflecting severe COVID-19^37,38,50^. Interestingly, most of the patients in our study also lack some of the clinical hallmarks of systemic cytokine storms like hypotension, capillary leakage syndrome and multi-organ failure indicating that a true cytokine storm still is relatively rare in COVID-19. This is further strengthened by the recent findings of mean IL-6 levels significant lover in COVID-19 with ARDS than bacterial sepsis with ARDS^51^. This suggests that only a minority of patients will benefit from specific anti-cytokine treatment. Thus, accurate patient selection is necessary, as cytokine blockers are potent drugs with risk of adverse reactions^52,53^. However, it would be of great importance to characterize and predict which patients that will progress to severe COVID-19 and could benefit from targeted therapy.

Several studies suggest that IL-6 may predict the risk of severe disease and mortality^37,40^. Beneficial effects of IL-6 inhibitors have also been shown in large observational studies^24,54,55^. Data from well-designed randomized clinical trials are critical to confirm a beneficial effect. Preliminary results from a clinical trial (*NCT04320615*) indicate that IL-6 inhibitor treatment could shorten time to discharge although there were no effects on mortality and clinical status^56^. Although our study doesn’t have enough numbers of patients to identify biomarkers for severity, our findings of the association between IL-6, IP-10, IL-1ra and RF in COVID-19 provides a foundation for further studies investigating their predictive value for RF.

There are some limitations in this study with a small sample size, especially for longitudinal samples where plasma could not be obtained on all time-points for several patients due to logistic challenges. Thus, small differences may not be detected or could give false positive results indicating that the results and significance should be interpreted with caution. The results from the ROC analyses were also all modest, therefore cut-off values and the cytokines predictive value must be interpreted with caution. In addition, associations may not necessarily reflect any causal relationship. Finally, plasma isolated from peripheral blood may not fully reflect the immune responses taking place in the infected tissue and more unbiased approaches will aid the characterization of the hyperinflammatory milieu in which several studies are emerging^14,57^.

In conclusion, we show that patients with RF, admitted to ICU and with biochemical signs of inflammation have increased cytokine production indicating a hyperinflammatory response, and increased level of IL-6, MCP-1, IP-10 and IL-1ra was associated with RF. Specific prognostic biomarkers can facilitate rapid detection of critically ill COVID-19 patients and aid in targeted treatment strategies, resulting in improved outcome of disease. Although we did not find convincing evidence of a true cytokine storm, especially combinations of IL-6 and MCP-1 may be further explored as potential biomarkers in severe COVID-19 infection.

## Supplementary Information

Supplementary Figure S1.
