# Supplementary material for: Increased interleukin-6 and macrophage chemoattractant protein-1 are associated with respiratory failure in COVID-19
Source: Sci Rep. 2020 Dec 10;10:21697. doi: 10.1038/s41598-020-78710-7 (PMC7729930; doi:10.1038/s41598-020-78710-7)
Supplement: Supplementary file 1 — Supplementary Figure S1. [file 41598_2020_78710_MOESM1_ESM.pdf]

Increased Interleukin-6 and Macrophage Chemoattractant Protein-1 are associated with Respiratory Failure in COVID-19

Marthe Jøntvedt Jørgensen[1,2], Jan Cato Holter[2,3], Erik Egeland Christensen[1,2], Camilla Schjalm[4], Kristian Tonby[1,2], Søren Erik Pischke[4,5], Synne Jenum[1], Linda G Skeie[1], Sarah Nur[1], Andreas Lind[3], Hanne Opsand[6], Tone Burvald Enersen[7], Ragnhild Grøndahl[8], Anne Hermann[7], Susanne Dudman[2,3], Fredrik Muller[2,3], Thor Ueland[2,9], Tom Eirik Mollnes[4,10,11] Pål Aukrust[2,9,14], Lars Heggelund[6,12], Aleksander Rygh Holten[2,13] , Anne Ma Dyrhol-Riise[1,2].

Online data supplement

Supplementary figure S1 longitudinal cytokine measurements during hospitalization. Cytokines measured in plasma for all patients at day of inclusion (n=30), day 2-5 (n=23) and day 7-10 (n=22) during hospitalization.

A

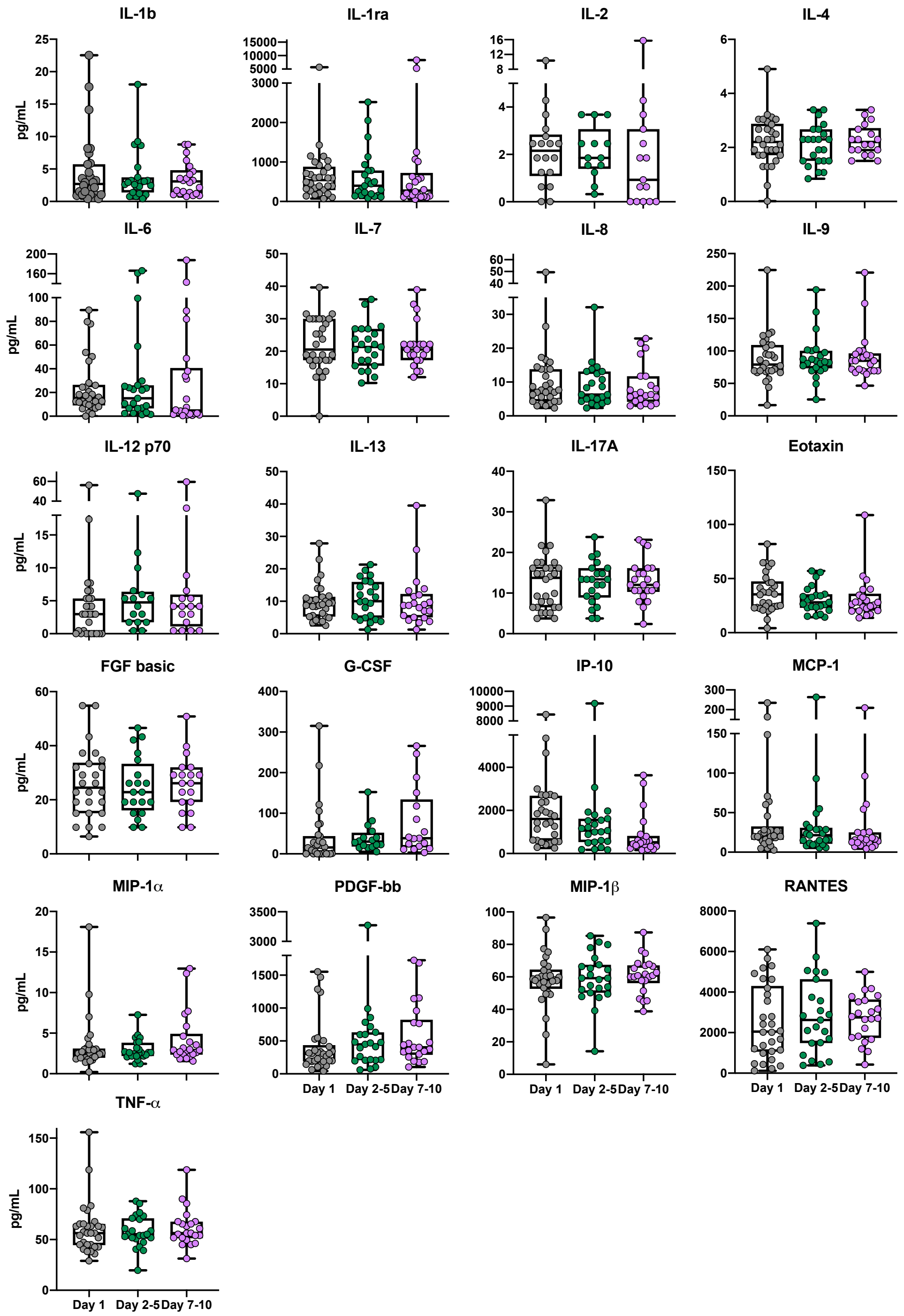
IL-1b
IL-1ra
IL-2
IL-4
IL-6
IL-7
IL-8
IL-9
IL-12 p70
IL-13
IL-17A
Eotaxin
FGF basic
G-CSF
IP-10
MCP-1
MIP-1α
PDGF-bb
MIP-1β
RANTES
TNF-α
pg/mL
Day 1
Day 2-5
Day 7-10
